# Socioeconomic inequalities in the quality of primary care under Brazil's national pay-for-performance programme: a longitudinal study of family health teams

**DOI:** 10.1016/S2214-109X(20)30480-0

**Published:** 2021-02-16

**Authors:** Roxanne Kovacs, Jorge O Maia Barreto, Everton Nunes da Silva, Josephine Borghi, Søren Rud Kristensen, Deivson Rayner T Costa, Luciano Bezerra Gomes, Garibaldi D Gurgel, Juliana Sampaio, Timothy Powell-Jackson

**Affiliations:** a Department of Global Health and Development, London School of Hygiene & Tropical Medicine, London, UK; b Oswaldo Cruz Foundation-Fiocruz, Brasília, Brazil; c Oswaldo Cruz Foundation-Fiocruz, Pernambuco, Brazil; d University of Brasília, Brasília, Brazil; e Centre for Health Policy, Institute of Global Health Innovation, Imperial College London, London, UK; f Danish Centre for Health Economics, University of Southern Denmark, Odense, Denmark; g Department of Health Promotion, Federal University of Paraiba, João Pessoa, Paraiba, Brazil; h Ministry of Health of Brazil, Brasília, Brazil

## Abstract

**Background:**

Many governments have introduced pay-for-performance programmes to incentivise health providers to improve quality of care. Evidence on whether these programmes reduce or exacerbate disparities in health care is scarce. In this study, we aimed to assess socioeconomic inequalities in the performance of family health teams under Brazil's National Programme for Improving Primary Care Access and Quality (PMAQ).

**Methods:**

For this longitudinal study, we analysed data on the quality of care delivered by family health teams participating in PMAQ over three rounds of implementation: round 1 (November, 2011, to March, 2013), round 2 (April, 2013, to September, 2015), and round 3 (October, 2015, to December, 2019). The primary outcome was the percentage of the maximum performance score obtainable by family health teams (the PMAQ score), based on several hundred (ranging from 598 to 914) indicators of health-care delivery. Using census data on household income of local areas, we examined the PMAQ score by income ventile. We used ordinary least squares regressions to examine the association between PMAQ scores and the income of each local area across implementation rounds, and we did an analysis of variance to assess geographical variation in PMAQ score.

**Findings:**

Of the 40 361 family health teams that were registered as ever participating in PMAQ, we included 13 934 teams that participated in the three rounds of PMAQ in our analysis. These teams were located in 11 472 census areas and served approximately 48 million people. The mean PMAQ score was 61·0% (median 61·8, IQR 55·3–67·9) in round 1, 55·3% (median 56·0, IQR 47·6–63·4) in round 2, and 61·6% (median 62·7, IQR 54·4–69·9) in round 3. In round 1, we observed a positive socioeconomic gradient, with the mean PMAQ score ranging from 56·6% in the poorest group to 64·1% in the richest group. Between rounds 1 and 3, mean PMAQ performance increased by 7·1 percentage points for the poorest group and decreased by 0·8 percentage points for the richest group (p<0·0001), with the gap between richest and poorest narrowing from 7·5 percentage points (95% CI 6·5 to 8·5) to –0·4 percentage points over the same period (–1·6 to 0·8).

**Interpretation:**

Existing income inequalities in the delivery of primary health care were eliminated during the three rounds of PMAQ, plausibly due to a design feature of PMAQ that adjusted financial payments for socioeconomic inequalities. However, there remains an important policy agenda in Brazil to address the large inequities in health.

**Funding:**

UK Medical Research Council, Newton Fund, and CONFAP (Conselho Nacional das Fundações Estaduais de Amparo à Pesquisa).

## Introduction

Primary health care is the linchpin of any health system but has long been neglected.^1^ The Astana Declaration 2018 sought to build momentum around a revitalised vision for primary health care.^2^ If political will is to turn into effective action, it is crucial that lessons are learned from country experience. Brazil has made substantial progress towards achieving universal health coverage, with the creation of a unified health system in 1990 (*Sistema Único de Saúde*).^3^ Over the past two decades, Brazil has invested heavily in primary health care, implementing innovative programmes at scale. The most high profile of these is the Family Health Strategy, the expansion of which is associated with reductions in mortality alongside reductions in racial inequalities in mortality.^4^, ^5^, ^6^, ^7^ In 2011, Brazil launched a national pay-for-performance (P4P) programme as part of its effort to strengthen primary health care through increased funding and improved organisational arrangements.

Health systems across the world have introduced P4P programmes to incentivise health providers to improve quality, use of care, and efficiency. Most of these programmes have focused on primary health care. Evaluations of P4P typically report average effects on these outcomes,^8^ with insufficient regard for the distribution of these effects. However, it is important to understand whether P4P programmes increase or reduce socioeconomic inequalities in health care. On one hand, P4P could exacerbate existing inequalities if primary care providers serving richer populations are better able to respond to the financial incentives. On the other, P4P could reduce inequalities if health providers serving poorer areas face larger potential rewards—either because they have greater scope for improvement or because the programme itself is designed to address inequalities.^9^, ^10^ The distributional effects of P4P across health-care providers or local areas remains unclear, with most of the limited evidence coming from the Quality and Outcomes Framework for general practices in the UK.^11^, ^12^, ^13^, ^14^

**Research in context**

**Evidence before this study**

We searched PubMed for articles investigating inequalities in the performance of providers participating in pay-for-performance (P4P) schemes, published from Jan 1, 1990, to May 29, 2020. The search strategy included terms related to P4P and inequalities: “(pay for performance OR P4P OR performance based financing OR results based financing OR performance based pay OR results based pay OR performance based contracting OR results based contracting) AND (inequ* OR equit* OR equality OR socio-economic OR socioeconomic OR disparity OR discrepancy OR fair* OR discrim*)”. We identified six studies, including one systematic review, that met our inclusion criteria. The majority of studies focused on the Quality and Outcomes Framework in the UK, showing that socioeconomic inequalities in clinical quality and other performance measures in the first year of the programme narrowed over time, as practices in the most deprived areas improved more than those in more affluent areas. There was only one study from a low-income setting. A study of P4P in Tanzania found that performance was initially higher among facilities serving wealthier populations, but these inequalities declined over time.

**Added value of this study**

To our knowledge, few studies exist that have examined inequalities in the performance of primary care providers under P4P in low-income and middle-income countries. We examined socioeconomic inequalities in the performance of family health teams under Brazil's National Programme for Improving Primary Care Access and Quality (PMAQ). We found that, initially, modest inequalities occurred in the delivery of primary health care on the basis of local area income, which were eliminated over successive rounds of PMAQ implementation. This was potentially due to a design feature that adjusted financial payments for socioeconomic inequalities.

**Implications of all the available evidence**

Existing evidence suggests that socioeconomic inequalities in the performance of primary care providers under P4P decreases over time—although a causal link is not established in the literature. The extent to which this is the case varies by setting. More evidence is needed on how P4P incentive design can be used to reduce existing socioeconomic inequalities within the health system.

Brazil's National Programme for Improving Primary Care Access and Quality (*Programa Nacional de Melhoria do Acesso e da Qualidade da Atenção Básica* [PMAQ]) was introduced in 2011. The aim of PMAQ was to improve access to and quality of primary care. The programme was explicitly designed, in its first 4 years, to address inequalities by adjusting the financial rewards given to family health teams (primary care providers) by differences in socioeconomic status between municipalities. PMAQ was one of the largest P4P programmes in the world, with about 40 000 family health teams participating and an expenditure of US$1·5 billion (R$8·6 billion) since its inception.^15^ Although literature on PMAQ is growing, previous studies have been limited to cross-sectional analyses of PMAQ at one point in time or to a narrow focus on specific outcomes, health conditions, or individual states.^16^, ^17^, ^18^, ^19^

In this study, we aimed to examine socioeconomic inequalities in the quality of care delivered by family health teams in successive rounds of PMAQ over the period 2011–19. We used programme data on the performance of family health teams nationwide, locating each team in a census area to establish the socioeconomic status of the catchment population. The primary analysis focused on family health teams providing care to over 48 million people.

## Methods

### Description of PMAQ

PMAQ was a federal programme that made financial payments to municipalities based on the performance of family health teams (appendix 2 pp 1–2). These teams are interdisciplinary, acting as the first point of primary health care in Brazil for a catchment population of about 3450 people per team. Each family health team is attached to a health facility and comprises at least one physician, nurse, nurse assistant, and full-time community health worker. As the decentralised administrative health authorities in Brazil, municipalities had autonomy in deciding how PMAQ funds were spent (consistent with budgetary rules based on federal laws). Although PMAQ funds had to be spent on health care, municipalities were not obligated to pass on funds as rewards to family health teams. PMAQ was implemented over three cycles: round 1 (November, 2011, to March 2013), round 2 (April, 2013, to September, 2015), and round 3 (October, 2015, to December, 2019). Participation in PMAQ was voluntary, with the proportion of municipalities opting into the programme increasing over time (71% in round 1, 91% in round 2, and 96% in round 3).^16^ Each round began with an assessment of the performance of family health teams, which determined the monthly payments made for the subsequent 2–3-year period of the round.

PMAQ incentivised hundreds of indicators (660 in round 3), some of which have changed across rounds (table 1). Indicators included those relating to structural quality of care (eg, availability of drugs and equipment), processes of care (eg, content of antenatal care and treatment completion rates), outcomes (eg, patient satisfaction, birthweight of children, and prevalence of chronic disease), utilisation of health care (eg, patient volume), and management processes (eg, proportion of appointments that are scheduled).^20^, ^21^, ^22^ PMAQ indicators were classified into three categories according to how they were measured: through self-assessment, routine monitoring, or external evaluation.

**Table 1:** Indicators and financial rewards in the design of PMAQ

	**Round 1 (2011–13)**	**Round 2 (2013–15)**	**Round 3 (2015–19)**
**Indicators of performance**			
Self-evaluation: FHTs reflect on their own performance	1 indicator (10% weight)	1 indicator (10% weight)	1 indicator (10% weight)
Monitoring: routine health management information system data on service utilisation and health outcomes submitted by FHT	24 indicators (20% weight)	20 indicators (20% weight)	11 indicators (30% weight)
External evaluation: data on quality of care, patient satisfaction, service utilisation, and management quality, collected via health-facility visits by external evaluators	573 indicators (70% weight)	893 indicators (70% weight)	648 indicators (60% weight)
**Financial reward system**			
Inequality adjustment	Yes	Yes	No
Performance groups	4 groups based on PMAQ scorerelative to performance of other teams within same socioeconomic band*	4 groups based on PMAQ scorerelative to performance of other teams within same socioeconomic band*	5 groups based on absolute PMAQ score†
**Financial reward per FHT, R$ per month**			
Worst	1700	1700	879
Worse	1700	1700	1758
Middle	NA	NA	4394
Better	5100	5100	7909
Best	8500	8500	8788

For the self-evaluation, teams received full points if they submitted the questionnaire, regardless of whether they assessed their own performance as being at a high or low level. The exchange rate was R$1=US$0·597 in 2011, R$1=US$0·491 in 2013, and R$1=US$0·379 in 2015. FHT=family health team. NA=not applicable. PMAQ=National Programme for Improving Primary Care Access and Quality.

* Groups based on score >1 SD lower than mean (group 1), <1 SD lower than mean (group 2), <1 SD higher than mean (group 3), and >1 SD higher than mean (group 4).

† Groups based on score 0–39 (group 1), 40–59 (group 2), 60–69 (group 3), 70–79 (group 4), and 80–100 (group 5).

For each indicator, a target was specified alongside the number of points awarded if the target was reached.^20^, ^21^, ^22^ To generate the PMAQ score for a family health team, the number of points achieved was divided by the number of points available in each of the three categories, and a weighted average was taken across the categories and multiplied by 100 (table 1). On the basis of the PMAQ score, each participating family health team was placed into a performance group that reflected the monthly financial reward. The amount of money each municipality received was the sum of the specific rewards of family health teams. In the first two rounds of PMAQ, an adjustment was made for socioeconomic inequality: municipalities in the country were divided into six socioeconomic bands, and performance groups were defined with reference to the distribution of PMAQ scores within each socioeconomic band. In round 3 of PMAQ, no adjustment was done for socioeconomic inequality, and performance groups were based solely on absolute PMAQ scores.

### Data sources

We drew on four sources of data. First, to capture team performance, we obtained the PMAQ scores of all family health teams from the Ministry of Health. The scores were based on data from the national routine health information system and data collected as part of the external evaluation, in which university-led survey teams visited every family health team. Second, to capture structural quality of care and basic characteristics of health facilities (to which family health teams are attached), we used survey data from the three rounds of the PMAQ external evaluation. Third, we used the 2010 Brazilian Population Census to measure the socioeconomic status of households in each census area (small geographical areas with roughly 5000 residents). The census also provided information on the geographical boundaries of each census sector (ie, the census area polygons). Finally, to identify the location of health facilities (to which family health teams are attached), we used their geographical coordinates (longitude and latitude) from the PMAQ external evaluation (round 3), supplemented by a health facility census done by the Ministry of Health in 2011.^23^

### Measures

Our primary measure of performance was the PMAQ score, calculated by the Ministry of Health, which we regard as a broad proxy for quality of care. The PMAQ score ranged from 0 to 100, with 0 representing a family health team with the lowest possible score and 100 representing a team with the highest possible score on all indicators. The score was interpreted as the percentage of the maximum score obtainable by a family health team. As previously mentioned, the set of indicators and formula used to calculate the PMAQ scores differed between rounds, leaving open the possibility that results could be an artifact of changes in measurement. We therefore developed a second measure of performance, based on a common set of indicators from each PMAQ round. These indicators captured the availability of 92 drugs, 23 items of equipment, and 22 consumables and diagnostic tests (appendix 2 p 2). This structural quality of care index was defined as the percentage of items that were available in each facility on the day of the external assessment visit.

We measured the socioeconomic status of each local area as the average monthly household income in each census sector (appendix 2 p 5). Using their geographical coordinates, we located family health teams within the polygon of their census area, allowing us to link the PMAQ score of family health teams to the mean income of households in their location. We also developed a broader measure of socioeconomic status, referred to as a vulnerability index, by combining census information on household income, literacy, and ethnic composition. Covariates included the proportion of the population younger than 5 years in each census area, the proportion of the population older than 50 years in each census area, the type of health facility to which the family health team was attached (health post, health centre, or other), and the number of clinical staff working at the facility.

### Statistical analysis

We analysed the PMAQ scores at the level of family health team. Unless specified, the analyses focused on family health teams that took part in all three rounds of PMAQ to avoid selection issues.

We first examined whether the PMAQ scores of family health teams were associated with the socioeconomic status of their local area (census sector). We grouped family health teams into ventiles (20 groups of equal size, with 697 family health teams in each group) on the basis of the census area income and plotted the mean PMAQ score in each ventile. We used a *t* test to calculate 95% CIs for the difference in the mean PMAQ scores between the poorest and richest areas. Tests of significance were based on a two-sided test. We regressed the PMAQ score on mean income of the census area, controlling for potential confounders (census area demographics and facility characteristics), using ordinary least squares regressions. We adjusted SEs for clustering by census level. We checked the sensitivity of our results using the structural quality of care index as a secondary measure of performance, with the vulnerability index as an alternative measure of socioeconomic status, and by including municipality-fixed effects in the regressions.

Finally, we examined geographical variation in PMAQ scores and did an analysis of variance to understand the extent to which states and municipalities explained variation in PMAQ scores. To visualise geographical variation in PMAQ scores, we mapped the data on all family health teams in each round using inverse distance weighting to interpolate values between team locations. We converted the measure of PMAQ performance into a Z score (by normalising to mean 0 and SD 1) because the scaling varied in each round. Analyses were done in Stata 16.1 SE and QGIS 3.10.10.

### Role of the funding source

The funder of the study had no role in study design, data collection, data analysis, data interpretation, or writing of the report. All authors had full access to all the data in the study. The corresponding author, as well as TP-J, JOMB, ENdS, and JB, had final responsibility for the decision to submit for publication.

## Results

Between November, 2011, and December, 2019, 40 361 family health teams were registered as participating in PMAQ and had their performance assessed in at least one round (17 482 in round 1, 30 523 in round 2, and 38 865 in round 3). Of these, 15 663 (38·8%) family health teams took part in all three rounds of the programme. Among these teams, 14 923 (95·3%) received a PMAQ score by the Ministry of Health (teams that did not submit all relevant data or meet basic requirements did not receive a score). Our analytical sample comprised 13 934 (93·4%) of 14 923 family health teams for which we had complete information on local area income and demographics and facility characteristics. These family health teams were located in 11 472 census areas, serving approximately 48 million people (appendix 2 p 6).

The mean PMAQ score—the percentage of the maximum score obtainable—was 61·0% (median 61·8, IQR 55·3–67·9) in round 1, 55·3% (56·0, 47·6–63·4) in round 2, and 61·6% (62·7, 54·4–69·9) in round 3 (table 2). The monthly household income across census areas containing the study family health teams was mean R$1470 (US$878) and median R$1320 (US$788).

**Table 2:** Sample description

		**Sample**
**Family health teams (n=13 934)**		
PMAQ score round 1		
	Mean (SD)	61·0 (9·9)
	Median (IQR)	61·8 (55·3–67·9)
PMAQ score round 2		
	Mean (SD)	55·3 (11·7)
	Median (IQR)	56·0 (47·6–63·4)
PMAQ score round 3		
	Mean (SD)	61·6 (12·6)
	Median (IQR)	62·7 (54·4–69·9)
**Facilities (n=11 750)**		
Health post		2345 (20%)
Health centre		8832 (75%)
Other		573 (5%)
Total clinical staff		
	Mean (SD)	15·9 (9·5)
	Median (IQR)	13·0 (11·0–17·0)
**Census areas (n=11 472)**		
Monthly household income, in thousands (R$)		
	Mean (SD)	1·47 (0·82)
	Median (IQR)	1·32 (0·9–1·82)
Percentage of census population younger than 5 years		
	Mean (SD)	7·3% (2·0)
	Median (IQR)	7·1% (5·9–8·5)
Percentage of census population older than 50 years		
	Mean (SD)	19·7% (6·4)
	Median (IQR)	19·1% (15·1–24·0)

Data are n (%), mean (SD), or median (IQR). PMAQ=National Programme for Improving Primary Care Access and Quality.

**Figure 1:**
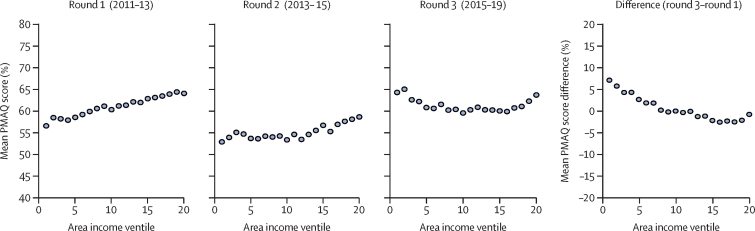
PMAQ score by ventile of local area household income Each graph shows the mean PMAQ score in 20 income groups (ventiles) with 697 family health teams in each group. Income groups are ranked from poorest (1) to richest (20). Mean monthly household income is $520 (US$310) in ventile 1, R$919 (US$549) in ventile 5, R$1357 (US$810) in ventile 10, R$1849 (US$1103) in ventile 15 and R$4039 (US$2409) in ventile 20. Exchange rate is for the year 2011. PMAQ=National Programme for Improving Primary Care Access and Quality.

We assessed the mean PMAQ score in each round across 20 income groups, ranked from poorest (1) to richest (20; figure 1). At the time of the round 1 assessment, we observed a positive socioeconomic gradient, whereby family health teams in richer areas achieved higher PMAQ scores than those in poorer areas. This gap was modest: the mean PMAQ score ranged from 56·6% in the poorest group (lowest ventile) to 64·1% in the richest group (highest ventile), with a difference of 7·5 percentage points (95% CI 6·5 to 8·5). By round 3, we observed no discernible socioeconomic gradient: the mean PMAQ score ranged from 63·7% in the poorest group to 63·3% in the richest group, with a difference of –0·4 percentage points (95% CI –1·6 to 0·8). Between rounds 1 and 3, mean PMAQ performance increased by 7·1 percentage points for the poorest group and decreased by 0·8 percentage points for the richest group (p<0·0001). The pattern of results was similar when we used a structural quality of care index as an alternative measure of family health team performance (appendix 2 p 7). As with the PMAQ score, the socioeconomic gradient became less steep over time, although a positive relationship between structural quality and local area income remained in round 3.

We used regression analyses to examine the relationship between PMAQ performance and local area income, controlling for characteristics of the health facility to which the family health team was attached and the local population (table 3). In round 1, teams located in poorer areas performed significantly worse than those in richer areas. A higher monthly household income of R$1000 (US$180) was associated with a 1·59 percentage point (95% CI 1·31–1·86; p<0·0001) higher PMAQ score. The association between PMAQ performance and income was weaker in round 2 and no longer significant in round 3. The change in the PMAQ score was negatively associated with income, confirming that the socioeconomic gradient became flatter over time (table 3).

**Table 3:** Association between PMAQ score and census area income

		**Round 1 (2011–13)**		**Round 2 (2013–15)**		**Round 3 (2015–19)**		**Difference (round 3–round 1)**	
		Coefficient (95% CI)	p value	Coefficient (95% CI)	p value	Coefficient (95% CI)	p value	Coefficient (95% CI)	p value
Monthly household income, in thousands (R$)		1·59 (1·31 to 1·86)	<0·0001	0·63 (0·36 to 0·90)	<0·0001	−0·21 (−0·51 to 0·10)	0·19	−1·79 (−2·18 to −1·40)	<0·0001
Proportion of census population younger than 5 years		−48·37 (−62·55 to −34·19)	<0·0001	−18·38 (−33·72 to −3·03)	0·019	−10·22 (−28·07 to 7·63)	0·26	38·15 (17·81 to 58·48)	0·0002
Proportion of census population older than 50 years		−9·7 (−13·92 to −5·49)	<0·0001	4·71 (−0·11 to 9·52)	0·055	1·35 (−3·94 to 6·65)	0·62	11·06 (5·01 to 17·10)	0·0003
Facility type*									
	Health centre	1·1 (0·63 to 1·56)	<0·0001	0·43 (−0·11 to 0·98)	0·12	0·65 (0·05 to 1·25)	0·035	−0·45 (−1·11 to 0·21)	0·18
	Other	1·79 (0·84 to 2·75)	0·0002	0·24 (−0·83 to 1·32)	0·66	0·75 (−0·41 to 1·90)	0·21	−1·05 (−2·28 to 0·19)	0·096
Total staff in facility		0·09 (0·07 to 0·11)	<0·0001	0·07 (0·05 to 0·09)	<0·0001	0·01 (−0·02 to 0·03)	0·60	−0·09 (−0·12 to −0·06)	<0·0001
Observations (teams)†		13 934	..	13 934	..	13 934	..	13 934	..
R^2^		0·06	..	0·02	..	<0·01	..	0·03	..

All models show results from ordinary least squares regressions. PMAQ=National Programme for Improving Primary Care Access and Quality.

* The reference group for facility type is health posts.

† Observations (teams) are clustered by census sector.

We obtained qualitatively similar results when we ran regressions with the structural quality index as the dependent variable, although the flattening of the socioeconomic gradient over time was less pronounced than with the PMAQ score (appendix 2 p 12). The results were not sensitive to the use of the full sample of family health teams rather than the panel (appendix 2 p 13) and our vulnerability index based on income, literacy, and ethnic composition rather than income alone (appendix 2 p 14). Regressions with municipality-fixed effects showed a significant association between income and PMAQ performance in the three rounds, which was small in magnitude (appendix 2 p 15).

**Figure 2:**
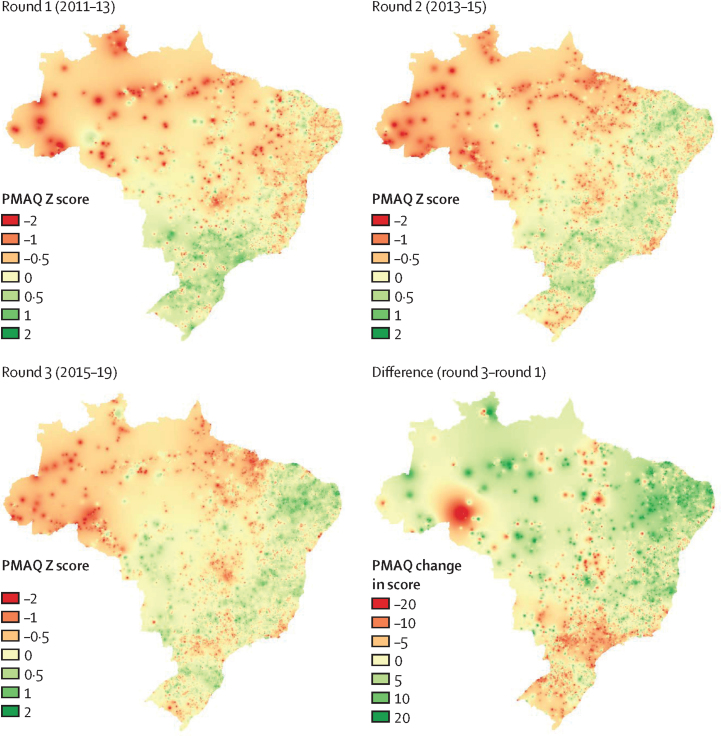
Geographical variation in PMAQ performance Red tones indicate lower Z scores (worse quality) or a decrease in the PMAQ score across rounds and green tones indicate higher Z scores (better quality) or an increase in the PMAQ score across rounds. PMAQ=National Programme for Improving Primary Care Access and Quality.

We assessed geographical variation in PMAQ performance for each round (figure 2). At the time of assessment in round 1, before bonus payments were awarded, family health teams in the more disadvantaged northern region had lower PMAQ scores than those in the wealthier southern region. In subsequent rounds, the geographical variation in performance changed: family health teams in the north and northeastern regions were no longer performing as poorly compared with the rest of the country as they were in the first round, whereas teams in the southern region and concentrated clusters of teams in the northern region started to perform less well compared with those in other parts of the country. These changes over time in the geographical distribution of the PMAQ scores are most clearly seen in the fourth map which shows the change in the score between round 1 and 3 (figure 2).

The analysis of variance revealed that states accounted for little of the variation in PMAQ scores (appendix 2 p 10). Municipalities tended to account for the majority of the variation, suggesting that municipality-level factors played an important role in determining family health team performance. The remaining variation in PMAQ scores was due to differences between family health teams within the same municipality, accounting for between 28–46% of the total variation. Between rounds of PMAQ, the variation between states accounted for a decreasing share of total variation, whereas variation within states and within municipalities accounted for an increasing share.

## Discussion

We investigated socioeconomic inequalities in the performance of family health teams that have participated in Brazil's national P4P programme since its introduction in 2011. Our analysis yielded three key results. First, we found that, in round 1, local area income was associated with better performance of family health teams throughout the income distribution. The strength of this relationship was modest, and these data most likely reflect pre-existing inequalities before PMAQ started, because data collection in round 1 was done before the disbursement of PMAQ funds to municipalities. Previous studies have documented income-related inequality in health care in Brazil, generally at one point in time.^24^, ^25^, ^26^ However, they focused on measures of health and utilisation, whereas we report on inequalities in the quality of primary care teams on the basis of a composite score generated from indicators of utilisation, structural quality, process of care, health outcomes, and managerial quality.

A second key result was that income inequality in the PMAQ score decreased over time, such that no relationship was found in the third round of programme implementation. The PMAQ score of family health teams in the bottom 5% of the local area income distribution improved over the three rounds, whereas those in the top 5% had no improvement. These results are in line with findings from a few comparable studies in other countries. Evidence from the Quality and Outcomes Framework for general practices in the UK shows that achievement across 48 indicators of clinical activity was initially skewed towards less deprived areas, but that this pro-rich bias was reduced over the first 3 years of the programme.^13^ A similar pattern of results was also found in Tanzania.^27^ A plausible explanation for the narrowing in the socioeconomic gap in PMAQ performance relates to the design of the scheme. Financial payments within PMAQ were adjusted for socioeconomic differences between municipalities in the first two rounds. This meant that poorer municipalities received higher rewards than they would otherwise have done that, if invested in primary care services, could be expected to reduce income-related inequalities in PMAQ performance. An alternative explanation that PMAQ led to a better distribution of resources to teams within municipalities—for example, by better targeting of under-performing teams in poorer areas—was not supported by the results from the regressions with municipality-fixed effects.

Our third key result was that, despite the modest (or absence of) income-related inequality, considerable geographical variation in PMAQ scores still occurred. Specifically, municipalities accounted for roughly half of the variation in PMAQ scores, suggesting that municipality factors are important determinants of family health team performance. Given the decentralised health system in Brazil and the fact that municipalities can raise and spend their own tax revenues, this is perhaps unsurprising. Municipalities have considerable autonomy in the implementation of health policy and are the recipient of PMAQ funds, with the authority to decide how money is spent. Such an interpretation is consistent with findings from a study of the Family Health Strategy in Brazil, which found that the programme was more effective in reducing mortality in municipalities with stronger health governance.^28^

An important consideration when interpreting the results is the extent to which patterns in the data reflect inequities in the provision of primary care. Equity is concerned with fairness. If more disadvantaged communities have greater health needs, socioeconomic equality in the performance of family health teams, as observed in the third round of PMAQ, would hide what most people would agree is an unfair distribution of health-care provision. Several studies have found that health-care provision in Brazil is inequitable, because households with low socioeconomic status and high health-care needs often have much less access to care than households with higher socioeconomic status and lower health-care needs.^29^, ^30^, ^31^, ^32^ Although we documented a reduction in inequality in the quality of service delivery, there still remains an important policy agenda in Brazil to address the large inequities in health.

This study has several strengths. We used newly available data on primary care performance for all family health teams participating in PMAQ. Additionally, we were able to link teams to a fine-grained measure of socioeconomic status at the census-sector level. Using a coarser measure would not have allowed us to capture the considerable income heterogeneity between neighbourhoods within municipalities. Finally, we were able to track the same facilities at three different points in time, allowing us to examine how the association between team performance and the socioeconomic status of each local area changed over time.

The study has several limitations. First, our main outcome of interest, the PMAQ score, has not been validated as a measure of quality and whether it is a predictor of health outcomes is unknown. By contrast, process quality indicators in some other P4P programmes, such as the Quality and Outcomes Framework in the UK, are closely linked to evidence on clinical effectiveness. However, the PMAQ score is the Brazilian Government's official measure of performance, was developed through a deliberate process with wide consultation, and was used to determine financial rewards in the programme. Second, some of the indicators used to generate the PMAQ score changed across rounds. Therefore, in principle, changes in the level and distributions of the score over time could have been an artifact of its measurement. Although we cannot rule out this possibility, it is reassuring that results were broadly similar when we used a structural quality of care index based on a common set of indicators over time. Third, we used a relatively crude measure of income, captured in the census through a few questions, rather than a detailed consumption and expenditure questionnaire. Nonetheless, results were robust to the use of our vulnerability index, a broader measure of socioeconomic status. Fourth, in linking family health teams to income data of their local area, we implicitly assumed that the household income of the census area in which a family health team was located reflected that of its catchment area, which might have extended beyond the census area. Fifth, although we used a measure of structural quality of care as a robustness check, such measures might not reflect the quality of clinical care actually received by patients. Sixth, when examining variation in PMAQ scores at different levels, an unknown share of the variation at the family health team level might be attributable to measurement error, possibly arising from within-observer variation (difference in scores by the same fieldworker assessing the same facility twice) or between-observer variation (difference in scores by two fieldworkers assessing the same facility). Finally, our analysis focused on the 13 934 family health teams that participated in all rounds of PMAQ to address potential selection issues. We urge caution in generalising the findings beyond the analytical sample (which is probably not representative of all PMAQ teams), although we note that the findings remained very similar when we used the full sample.

This study makes an important contribution to the literature on the distributional consequences of P4P schemes. Our results suggest that, within one of the largest P4P schemes in the world, socioeconomic inequalities in the performance of primary care providers decreased over the course of the programme. This was potentially due to a design feature that adjusted financial payments for socioeconomic inequalities. Further research is needed on the distributional consequences of P4P schemes in different settings, particularly in low-income and middle-income countries, and for schemes with different incentive designs.

For the **London School of Hygiene & Tropical Medicine Data Compass** see https://datacompass.lshtm.ac.uk/2016/

## Data sharing

The data used in this study are owned by the Ministry of Health of Brazil, which should be contacted by those who wish to use the data. The analysis code for the paper will be made available on the London School of Hygiene & Tropical Medicine's Data Compass.

## Supplementary Materials

Portuguese translation of the abstract
