# Supplementary material for: Socioeconomic inequalities in the quality of primary care under Brazil's national pay-for-performance programme: a longitudinal study of family health teams
Source: Lancet Glob Health. 2021 Feb 16;9(3):e331–9. doi: 10.1016/S2214-109X(20)30480-0 (PMC7900523; doi:10.1016/S2214-109X(20)30480-0)
Supplement: Portuguese translation of the abstract [file mmc1.pdf]

THE LANCET
Global Health

Supplementary appendix 1

This translation in Portuguese was submitted by the authors and we reproduce it as supplied. It has not been peer reviewed. The Lancet's editorial processes have only been applied to the original in English, which should serve as reference for this manuscript.

Esta tradução em português foi submetida pelos autores e nós não fizemos quaisquer alterações. Esta versão não foi revista por pares. O processo editorial do The Lancet só foi aplicado à versão original em inglês, que deve servir como referência para este artigo.

Supplement to: Kovacs R, Maia Barreto JO, Nunes da Silva E, et al. Socioeconomic inequalities in the quality of primary care under Brazil's national pay-for-performance programme: a longitudinal study of family health teams. *Lancet Glob Health* 2021; **9:** e331–39.

Desigualdades socioeconômicas na qualidade da atenção primária sob o programa nacional de pagamento por desempenho do Brasil: um estudo longitudinal das equipes de saúde da família

Sumário

Contexto Muitos governos têm introduzido programas de pagamento por desempenho para incentivar os provedores a melhorar a qualidade dos cuidados em saúde. Evidências sobre se esses programas reduzem ou aumentam desigualdades nesses cuidados são escassas. Neste estudo, foram avaliadas desigualdades socioeconômicas no desempenho das equipes de saúde da família que aderiram ao Programa Nacional de Melhoria do Acesso e Qualidade da Atenção Básica (PMAQ) do Brasil.

Métodos Para este estudo longitudinal, foram analisados dados sobre a qualidade do cuidado prestado pelas equipes de saúde da família que participaram dos três ciclos de implementação do PMAQ: ciclo 1 (novembro de 2011 a março de 2013), ciclo 2 (abril de 2013 a setembro de 2015), e ciclo 3 (outubro de 2015 a dezembro de 2019). O desfecho principal foi o percentual da nota máxima de desempenho obtida pelas equipes de saúde da família (a nota do PMAQ), baseada em centenas de indicadores (variando de 598 a 914) relacionados com o cuidado ofertado. Usando dados de renda domiciliar vinculados aos setores censitários, a nota do PMAQ foi examinada em 20 estratos de percentis de renda. Uma regressão por mínimos quadrados ordinários foi usada para examinar a associação entre as notas do PMAQ e a renda dos setores censitários, ao longo nos ciclos de implementação, e uma análise de variância foi aplicada para investigar variação geográfica na nota do PMAQ.

Resultados Das 40 361 equipes de saúde da família que participaram do PMAQ em algum momento, 13 934 equipes que participaram dos três ciclos do PMAQ foram incluídas na análise. Essas equipes estavam localizadas em 11 472 setores censitários e proveram cobertura para 48 milhões de pessoas. A nota média do PMAQ foi 61,0% (mediana 61,8, IQR 55,3–679) no ciclo 1, 55,3% (mediana 56,0, IQR 47,6–63,4) no ciclo 2, e 61,6% (mediana 62,7, IQR 54,4–69,9) no ciclo 3. No ciclo 1, foi observado um gradiente socioeconômico positivo, com a média da nota do PMAQ variando de 56,6% no grupo mais pobre a 64,1% no grupo mais rico. Entre os ciclos 1 e 3, a média do desempenho do PMAQ aumentou 7,1 pontos percentuais para o grupo mais pobre e diminuiu 0,8 ponto percentual para o grupo mais rico ($p<0,0001$), com a diferença entre o grupo mais rico e o mais pobre diminuindo de 7,5 pontos percentuais (95% IC 6,5–8,5) para -0,4 ponto percentual no mesmo período (–1,6 a 0,8).

Interpretação As desigualdades de renda existentes na oferta dos cuidados primários de saúde foram eliminadas durante os três ciclos do PMAQ, possivelmente devido às características do desenho do PMAQ, que ajustava pagamentos financeiros por desigualdades socioeconômicas. Entretanto, ainda persiste uma agenda política importante para enfrentar as grandes iniquidades em saúde.

Financiamento UK Medical Research Council, Newton Fund, e CONFAP (Conselho Nacional das Fundações Estaduais de Amparo à Pesquisa).

Socioeconomic inequalities in the quality of primary care under Brazil's national pay-for-performance programme: a longitudinal study of family health teams

Summary

Background Many governments have introduced pay-for-performance programmes to incentivise health providers to improve quality of care. Evidence is on whether these programmes reduce or exacerbate disparities in health care is scarce. In this study, we aimed to assess socioeconomic inequalities in the performance of family health teams under Brazil's National Programme for Improving Primary Care Access and Quality (PMAQ).

Methods For this longitudinal study, we analysed data on the quality of care delivered by family health teams participating in PMAQ over three rounds of implementation: round 1 (November 2011, to March 2013), round 2(April 2013, to September 2015), and round 3 (October 2015, to December 2019). The primary outcome was the percentage of the maximum performance score obtainable by family health teams (the PMAQ score), based on several hundred (ranging from 598 to 914) indicators of health-care delivery. Using census data on household income of local areas, we examined the PMAQ score by income ventile. We used ordinary least squares regressions to examine the association between PMAQ scores and the income of each local area across implementation rounds, and we did an analysis of variance to assess geographical variation in PMAQ score.

Findings Of the 40 361 family health teams that were registered as ever participating in PMAQ, we included13 934 teams that participated in the three rounds of PMAQ in our analysis. These teams were located in 11 472 census areas and served approximately 48 million people. The mean PMAQ score was 61·0% (median 61·8, IQR 55·3–67·9)in round 1, 55·3% (median 56·0, IQR 47·6–63·4) in round 2, and 61·6% (median 62·7, IQR 54·4–69·9) in round 3.In round 1, we observed a positive socioeconomic gradient, with the mean PMAQ score ranging from 56·6% in the poorest group to 64·1% in the richest group. Between rounds 1 and 3, mean PMAQ performance increased by7·1 percentage points for the poorest group and decreased by 0·8 percentage points for the richest group ($p<0·0001$),with the gap between richest and poorest narrowing from 7·5 percentage points (95% CI 6·5–8·5) to –0·4 percentage points over the same period (–1·6 to 0·8).

Interpretation Existing income inequalities in the delivery of primary health care were eliminated during the three rounds of PMAQ, plausibly due to a design feature of PMAQ that adjusted financial payments for socioeconomic inequalities. However, there remains an important policy agenda in Brazil to address the large inequities in health.

Funding UK Medical Research Council, Newton Fund, and CONFAP (Conselho Nacional das Fundações Estaduais deAmparo à Pesquisa).
