# Supplementary material for: Socioeconomic inequalities in the quality of primary care under Brazil's national pay-for-performance programme: a longitudinal study of family health teams
Source: Lancet Glob Health. 2021 Feb 16;9(3):e331–9. doi: 10.1016/S2214-109X(20)30480-0 (PMC7900523; doi:10.1016/S2214-109X(20)30480-0)
Supplement: Supplementary appendix 2 [file mmc2.pdf]

THE LANCET
Global Health

Supplementary appendix 2

This appendix formed part of the original submission and has been peer reviewed. We post it as supplied by the authors.

Supplement to: Kovacs R, Maia Barreto JO, Nunes da Silva E, et al. Socioeconomic inequalities in the quality of primary care under Brazil's national pay-for-performance programme: a longitudinal study of family health teams. *Lancet Glob Health* 2021; **9:** e331–39.

A1 – Description of PMAQ

Brazil is amongst the most unequal countries in the world. Income inequality is high, as reflected by a GINI coefficient of 0.53 in 2015.[1] Health inequalities are substantial – individuals from disadvantaged socioeconomic groups have less access to primary care[2,3] and mental health services[4], and suffer a higher prevalence of non-communicable diseases[5]. There are also substantial social inequalities in health behaviours, with rates of smoking and sedentary lifestyle higher among disadvantaged groups.[6]

PMAQ was a federal programme that made financial payments to municipalities based on the performance of family health teams. These teams are interdisciplinary, acting as the first point of primary healthcare in Brazil for a catchment population of around 3,450 people. Each family health team is attached to a health facility (there are on average 1.3 family health teams per facility), and comprises at least a physician, a nurse, a nurse assistant, and a full-time community health agent. As the decentralised administrative health authority in Brazil, municipalities had autonomy in deciding how PMAQ funds are spent (as long as this complied with budgetary rules based on federal laws). Although PMAQ funds had to be spent on healthcare, municipalities were not obligated to pass on funds as rewards to family health teams. . PMAQ was implemented over three cycles: round 1 (Nov 2011 – Mar 2013), round 2 (Apr 2013 – Sept 2015) and round 3 (Oct 2015 – Dec 2019). Participation in PMAQ was voluntary, with the proportion of municipalities opting into the programme increasing over time (71% in round 1, 91% in round 2, and 96% in round 3). Each round began with an assessment of the performance of family health teams, which determines the monthly financial payments made for the subsequent two to three-year period of the round.

PMAQ incentivised a large number of indicators (660 in round 3), some of which have changed across rounds (appendix p.9). The complete list of indicators can be found in Ministry of Health policy documents.[7–9] Indicators were selected through workshops involving the Ministry of Health, researchers, and health managers at the municipal and state levels. Indicators included those relating to structural quality of care (e.g. availability of drugs and equipment), processes of care (e.g. content of antenatal care, treatment completion rates), outcomes (e.g. patient satisfaction, birth weight of children, prevalence of chronic disease), utilisation of healthcare (e.g. patient volume) and management processes (e.g. proportion of appointments that are scheduled). PMAQ indicators were classified into three categories according to how they were measured: through self-assessment; routine monitoring; and external evaluation (appendix p.9).

For each indicator, a target was specified alongside the number of points awarded if the target is reached.[7–9] To generate the PMAQ score for a family health team, the number of points achieved was divided by the number of points available in each of the three categories, a weighted average was taken across the categories, and multiplied by 100 (appendix p.9). Based on the PMAQ score, each participating family health team was placed into a performance group that reflects the monthly financial reward. The amount of money each municipality received was the sum of the family health team-specific rewards. In the first two rounds of PMAQ, there was an adjustment for socioeconomic inequality – municipalities in the country were divided into six socioeconomic bands, and performance groups were defined with reference to the distribution of PMAQ scores within each socioeconomic band. For example, teams within the same municipality socioeconomic band that performed one standard deviation above the mean received the largest financial reward. In round 3 of PMAQ, there was no adjustment for socioeconomic inequality – performance groups were based solely on absolute PMAQ scores. To our knowledge, no official reason was given for why the financial adjustment for socioeconomic status was dropped.

A2 – Structural quality index

The structural quality index captures the availability of 92 drugs, 23 items of equipment, and 22 consumables and diagnostic tests, which were included in the external evaluation questionnaire in all three PMAQ rounds. The full list of items is shown below.

Drugs:

- Aluminum Hydroxide
- Metoclopramide Hydrochloride
- Ranitidine Hydrochloride
- Omeprazole
- Amoxicillin
- Ammoxicillin + Potassium Clavulanate
- Azithromycin
- Procaine Benzylpenicillin + Potassium Benzylpenicillin
- Cephalexin (Sodium or Hydrochloride)
- Ciprofloxacin Hydrochloride
- Ketoconazole
- Clarithromycin
- Chloramphenicol
- Clindamycin Hydrochloride
- Erythromycin Stearate
- Gentamicin Sulphate
- Nitrofurantoin
- Sulfadiazine
- Tetracycline Hydrochloride
- Fluconazole
- Itraconazole
- Miconazole Nitrate
- Nystatin
- Carbamazepine
- Clonazepam
- Phenytoin Sodium
- Phenobarbital
- Lithium Carbonate
- Fluoxetine Hydrochloride
- Sodium Valproate or Valproic Acid

- Nortriptyline Hydrochloride
- Haloperidol
- Biperiden Hydrochloride
- Chlorpromazine Hydrochloride
- Clomipramine Hydrochloride
- Amitriptyline Hydrochloride
- Diazepam
- Albendazole
- Metronidazole
- Teclozana
- Ivermectin
- Permethrin
- Spamycin
- Beclomethasone Dipropionate
- Ipratropium Bromide
- Prednisone
- Salbutamol Sulphate
- Norethisterone Enanthate and Estradiol Valerate
- Ethinyl Estradiol and Levonorgestrel
- Levonorgestrel
- Medroxyprogesterone Acetate
- Norethisterone
- Estriol Vaginal Cream
- Conjugated Estrogens
- Insulin
- Amlodipine Besylate
- Atenolol
- Metoprolol Succinate
- Propranolol Hydrochloride
- Captopril
- Enalapril Maleate
- Hydralazine Hydrochloride
- Spironolactone
- Furosemide
- Hydrochlorothiazide
- Verapamil Hydrochloride
- Amiodarone Hydrochloride
- Propafenone Hydrochloride
- Acetylsalicylic Acid
- Simvastatin
- Digoxin
- Potassium Losartan
- Calcium Carbonate and Cholecalciferol
- Alendronate Sodium Enough
- Sodium Dipyrone
- Ibuprofen
- Acetaminophen
- BCG vaccine
- Viral Triple vaccine
- DTP vaccine
- Human Rotavirus vaccine
- DTP adult vaccine
- Yellow Fever vaccine
- Seasonal Influenza vaccine
- Hepatitis B vaccine
- Meningococcal C vaccine
- Pneumococcal 10 vaccine
- Ferrous Sulphate
- Folic Acid
- Pyridoxine Hydrochloride
- Thiamine
- Retinol Palmitate

<u>Equipment:</u>

- Infant scales
- Measuring tape (adults)
- Adult stethoscope
- Patriotic stethoscope
- Spotlights for gynaecological examination
- Fridge for vaccines
- Fridge for drugs
- Glucometer
- Clinical Flashlight
- Sphygmomanometer
- Gynaecological examination table
- Table/stretcher for clinical examination
- Ophthalmoscopes
- Otoscopes
- Esthesiometer
- Pinard horn
- Microscope
- Thermometer with linear cable

- Clinical thermometer
- Paediatric sphygmomanometer
- Nebuliser
- Anthropometric scales 150 kg
- Anthropometric scales 200 kg

Consumables and tests:

- Tongue depressor
- Ayres spatula
- Adhesive tape
- Fixator spray
- Gauze
- Frosted glass blade
- Capillary blood glucose measuring reagents
- Disposable syringes of various sizes
- Disposable syringes with needle attached
- Disposal containers for sharp objects
- Slides
- Bandages
- Vaccine coolers
- Tape
- Disposable Speculum
- Macrobeads
- Endocervical brush
- Disposable needles of various sizes
- Male Condom
- Female condom
- Rapid syphilis test
- Rapid pregnancy test
- Rapid HIV test
- Thick blood smear test

A3 – Census sector income

In the 2010 census, respondents who have worked for at least one hour during the week of the 25th of July 2010, are asked the following questions to assess their income:

- In your primary place of work, what monthly gross income did you earn in July 2010? (*No trabalho principal, qual era o rendimento bruto (ou a retirada) mensal que ganhava habitualmente em julho de 2010?*)
- For any other work, what monthly gross income did you earn in July 2010? (*Nos demais trabalhos, qual era o rendimento bruto (ou a retirada) mensal que ganhava habitualmente em julho de 2010?*)

A4 – Vulnerability index

To capture the socio-economic status of households living in each census sector, we create a vulnerability index. The index captures: monthly household income $(income)$, the proportion of the population aged 15 (age_{15}) and over who are literate and the proportion of the population who are white $(white)$. These variables were used as they are indicators of social risk factors relevant to the Brazilian setting and were available in the 2010 census [1,2].

We first standardise $income, age_{15}$ and $white,$ by subtracting the mean from each observation and dividing by the standard deviation, creating $income_{std}, age_{15_{std}}$ and $white_{std}$. To create the index, we take a simply unweighted average of each component $\frac{(income_{std}+age_{15_{std}}+white_{std})}{3}$, we then standardise again by subtracting the mean from each observation and dividing by the standard deviation. The resulting index has a mean of zero and a standard deviation of one. We divide census sectors into 20 equally sized groups (ventiles) based on their socio-economic status to mirror the analysis conducted with monthly household income.

A5 – Figures

Figure A1. Study flow diagram

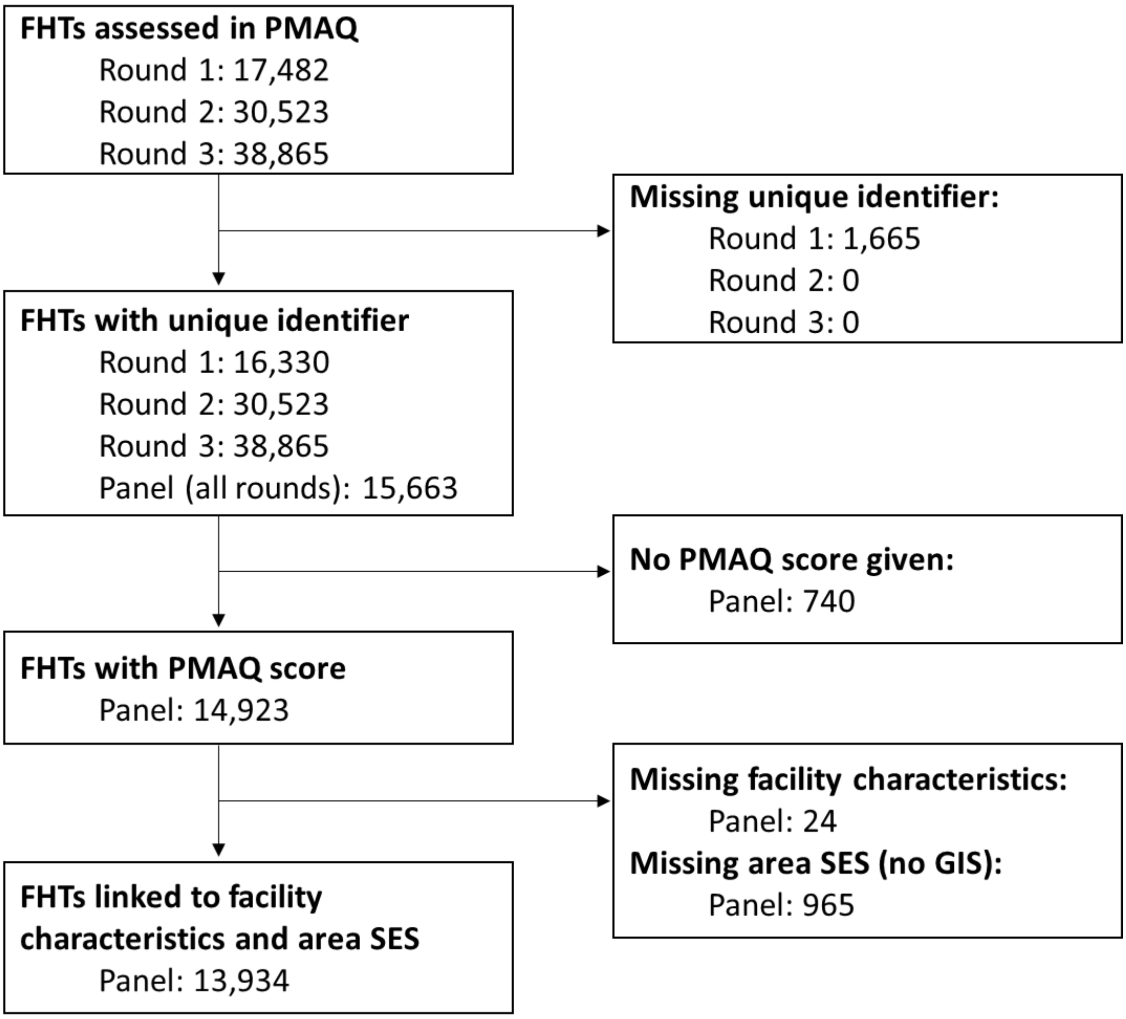

Note: Family health teams that were not awarded a PMAQ score either failed to submit data needed to calculate the PMAQ score (say, DHIS data for the monitoring indicators) or did not have items of equipment deemed essential by the MOH (such as a dental chair).

Figure A2. Structural quality of care index by ventile (20 groups) of mean monthly household income of local area

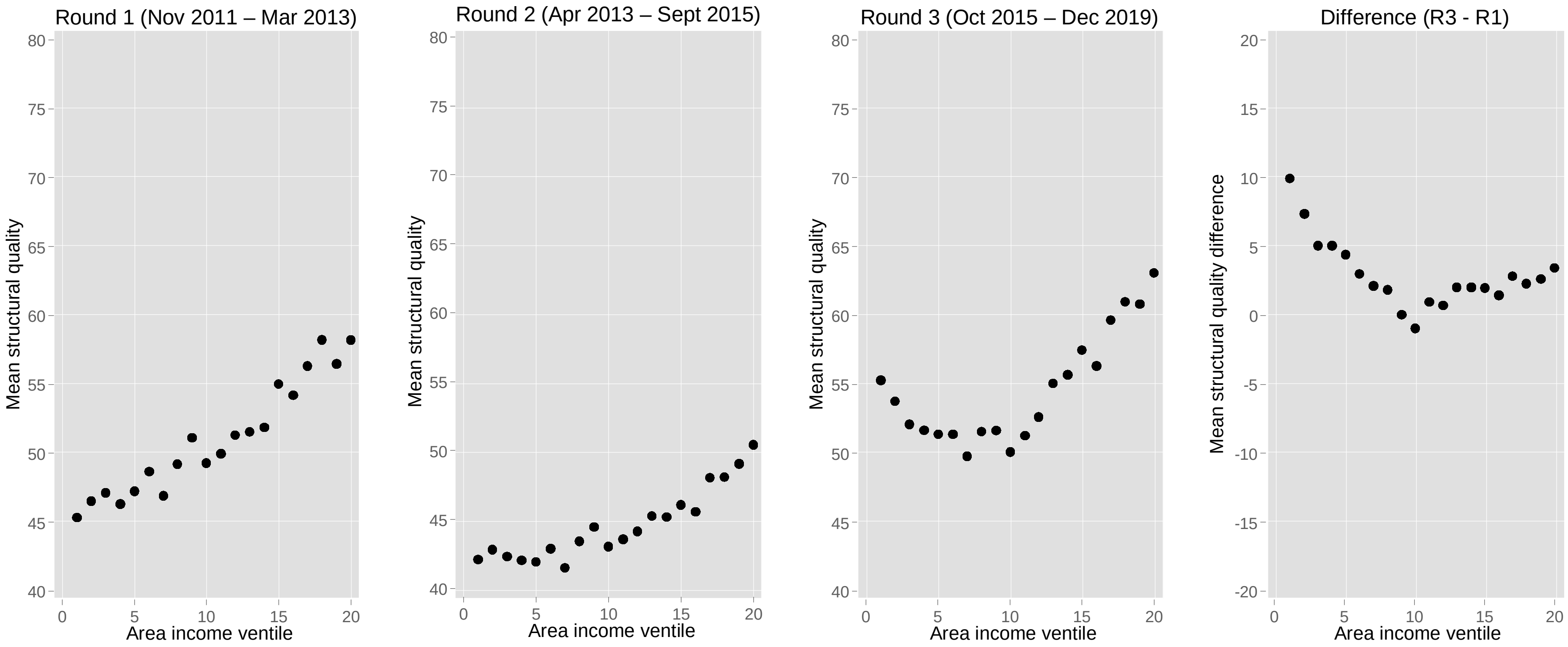

Figure A3. PMAQ score by ventile (20 groups) of vulnerability index of local area

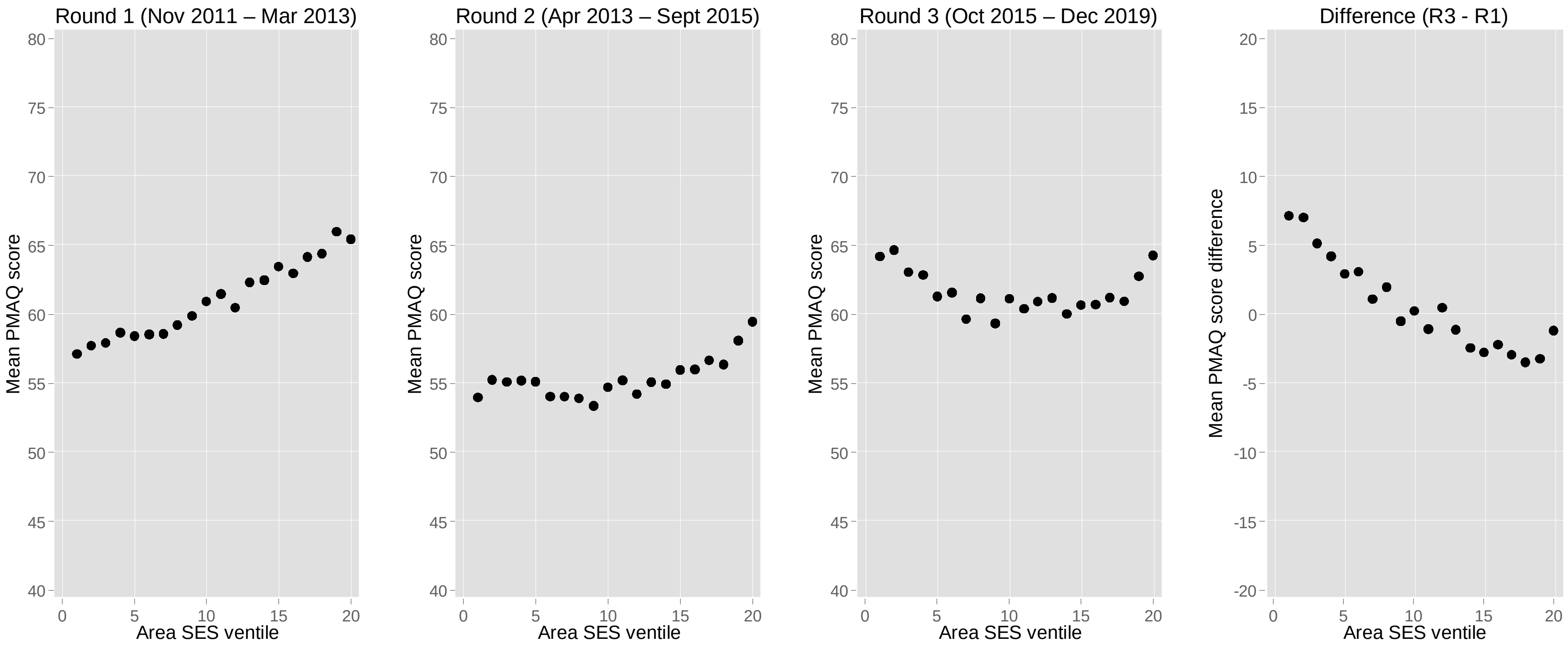

Figure A4. PMAQ score by ventile (20 groups) of mean monthly household income of local area for all family health teams

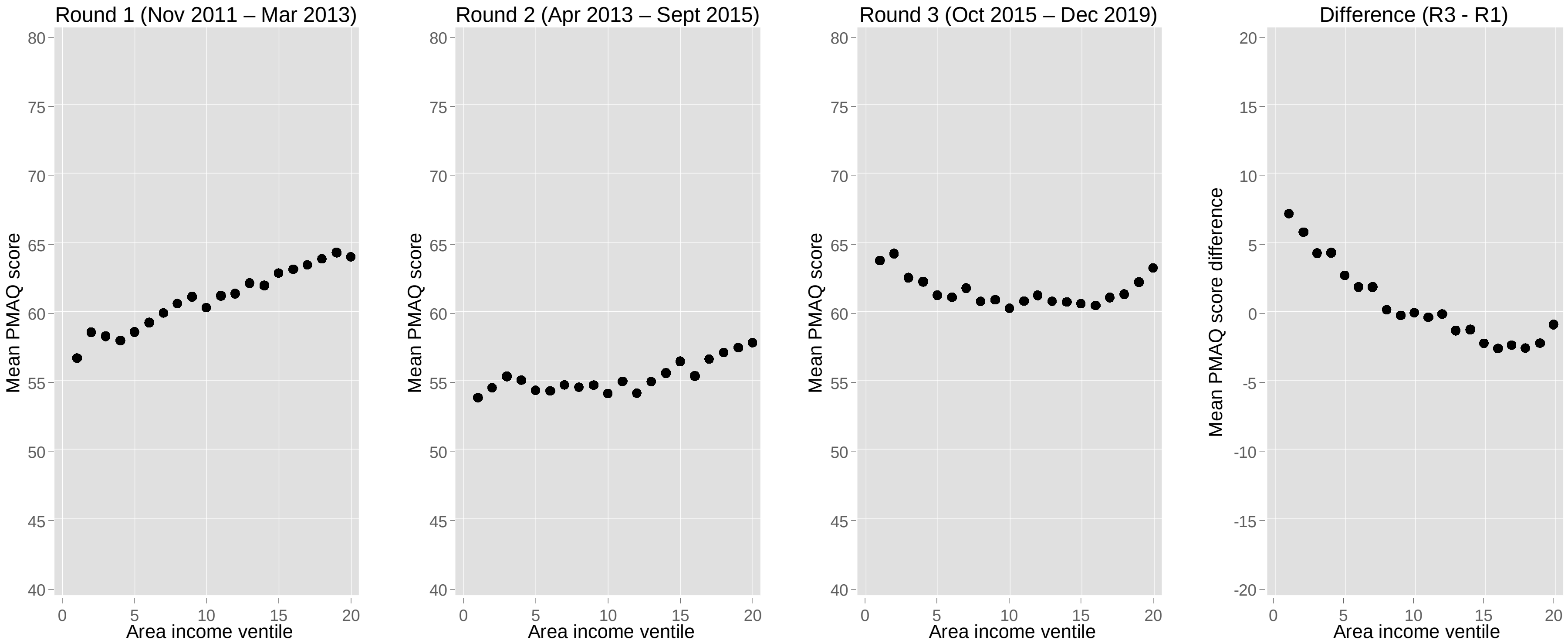

A6 - Tables

Table A1. Source of geographical variation in PMAQ performance

	Between states	Within states, between municipalities	Within municipalities (between family health teams)
Round 1 (Nov 2011 – Mar 2013)	18.2%	53.5%	28.3%
Round 2 (Apr 2013 – Sep 2015)	11.1%	49.4%	39.5%
Round 3 (Oct 2015 – Dec 2019)	9.7%	45.5%	44.8%
Difference (Round 3 – Round 1)	12.9%	40.8%	46.3%

Notes: Family health teams per municipality has a mean of 3.98 and a median of 2. The sample size is 13,934.

Table A2. Descriptive statistics of structural quality index

	mean (SD)	median (IQR)
Facilities (N=10,358)		
Structural quality index round 1	48.66 (16.83)	49.64 (34.31 - 59.85)
Structural quality index round 2	42.73 (12.41)	44.53 (32.12 - 51.82)
Structural quality index round 3	51.78 (18.02)	54.41 (33.82 - 64.71)

Table A3. Census area monthly household income

	Observations	Median	Mean	SD	25th pct.	75th pct.
Areas with PMAQ family health team in panel	11,472	1,323.12	1,473.88	816.79	903.05	1,821.29
Areas with family health team in round 3 only	15,291	1,213.71	1,380.92	833.26	790.97	1,728.55
Areas without a PMAQ family health team	277,533	1,564.22	2,104.48	2,075.39	1,035.82	2,360.61

Table A4. Structural quality index of facilities based on average monthly household income of the local area in (R$ 1,000)

	Round 1 (Nov 2011 – Mar 2013)		Round 2 (Apr 2013 – Sept 2015)		Round 3 (Oct 2015 – Dec 2019)		Difference (Round 3 – Round 1)	
	Coef (95% CI)	P value	Coef (95% CI)	P value	Coef (95% CI)	P value	Coef (95% CI)	P value
Monthly household income (in R$ 1,000)	1.93 (1.48 to 2.38)	0.000	1.07 (0.75 to 1.38)	0.000	0.98 (0.50 to 1.46)	0.000	-0.95 (-1.40 to -0.49)	0.000
Proportion of census population under 5	-3.36 (-24.79 to 18.07)	0.758	3.61 (-12.85 to 20.08)	0.667	24.66 (1.15 to 48.17)	0.040	28.02 (5.15 to 50.89)	0.016
Proportion of census population over 50	-2.39 (-9.45 to 4.67)	0.507	-6.63 (-11.89 to -1.37)	0.014	5.93 (-1.80 to 13.65)	0.133	8.31 (1.25 to 15.38)	0.021
Facility type (health post)								
Health centre	2.17 (1.40 to 2.95)	0.000	0.37 (-0.20 to 0.95)	0.205	0.69 (-0.14 to 1.51)	0.102	-1.49 (-2.31 to -0.67)	0.000
Other	1.31 (-0.32 to 2.94)	0.115	1.07 (-0.15 to 2.29)	0.085	-0.42 (-2.12 to 1.28)	0.631	-1.73 (-3.41 to -0.05)	0.043
Total staff in facility	0.44 (0.40 to 0.49)	0.000	0.31 (0.28 to 0.33)	0.000	0.41 (0.38 to 0.45)	0.000	-0.03 (-0.07 to 0.01)	0.184
Observations (teams)	10,358		10,358		10,358		10,358	
R-squared	0.07		0.06		0.05		0.01	

Notes: All models show results from OLS regressions. 95% confidence intervals are shown in brackets. Observations (facilities) are clustered by census sector. Monthly household income is shown in terms of R$ 1,000. The comparison group for facility type is “Health posts”.

Table A5. Association between PMAQ score and census area income for all family health teams

	Round 1 (Nov 2011 – Mar 2013)		Round 2 (Apr 2013 – Sept 2015)		Round 3 (Oct 2015 – Dec 2019)		Difference (Round 3 – Round 1)	
	Coef (95% CI)	P value	Coef (95% CI)	P value	Coef (95% CI)	P value	Coef (95% CI)	P value
Monthly household income (in R$ 1,000)	1.52<br>(1.25 to 1.79)	0.000	0.17<br>(-0.05 to 0.39)	0.138	-0.65<br>(-0.95 to -0.36)	0.000	-1.79<br>(-2.18 to -1.40)	0.000
Proportion of census population under 5	-48.07<br>(-61.93 to -34.21)	0.000	-56.34<br>(-68.07 to -44.61)	0.000	-46.79<br>(-60.39 to -33.19)	0.000	38.15<br>(17.81 to 58.48)	0.000
Proportion of census population over 50	-9.55<br>(-13.68 to -5.42)	0.000	1.96<br>(-1.74 to 5.66)	0.300	8.38<br>(4.16 to 12.61)	0.000	11.06<br>(5.01 to 17.10)	0.000
Facility type (health post)								
Health centre	1.20<br>(0.74 to 1.66)	0.000	0.90<br>(0.48 to 1.31)	0.000	0.94<br>(0.48 to 1.40)	0.000	-0.45<br>(-1.11 to 0.21)	0.181
Other	1.87<br>(0.92 to 2.81)	0.000	0.92<br>(0.18 to 1.66)	0.015	1.15<br>(0.27 to 2.03)	0.010	-1.05<br>(-2.28 to 0.19)	0.096
Total staff in facility	0.10<br>(0.08 to 0.12)	0.000	0.08<br>(0.06 to 0.10)	0.000	-0.03<br>(-0.05 to -0.00)	0.024	-0.09<br>(-0.12 to -0.06)	0.000
Observations (teams)	14,704		27,157		33,601		13,934	
R-squared	0.06		0.02		0.01		0.03	

Notes: All models show results from OLS regressions. 95% confidence intervals are shown in brackets Observations (teams) are clustered by census sectors. Monthly household income is shown in terms of R$ 1,000. The reference group for facility type is “health posts”.

Table A6. PMAQ score of family health teams based on vulnerability index of local area

	Round 1 (Nov 2011 – Mar 2013)		Round 2 (Apr 2013 – Sept 2015)		Round 3 (Oct 2015 – Dec 2019)		Difference (Round 3 – Round 1)	
	Coef (95% CI)	P value	Coef (95% CI)	P value	Coef (95% CI)	P value	Coef (95% CI)	P value
Vulnerability index	2.39<br>(2.18 to 2.60)	0.000	0.68<br>(0.42 to 0.93)	0.000	-0.62<br>(-0.88 to -0.35)	0.000	-3.01<br>(-3.32 to -2.70)	0.000
Proportion of census population under 5	-21.30<br>(-35.54 to -7.06)	0.003	-13.59<br>(-29.31 to 2.14)	0.090	-20.42<br>(-38.74 to -2.10)	0.029	0.88<br>(-19.63 to 21.39)	0.933
Proportion of census population over 50	-9.62<br>(-13.75 to -5.50)	0.000	4.86<br>(0.06 to 9.66)	0.047	1.48<br>(-3.81 to 6.77)	0.584	11.10<br>(5.14 to 17.06)	0.000
Facility type (health post)								
Health centre	0.78<br>(0.32 to 1.24)	0.001	0.37<br>(-0.17 to 0.92)	0.180	0.76<br>(0.16 to 1.36)	0.013	-0.01<br>(-0.66 to 0.64)	0.967
Other	1.23<br>(0.31 to 2.15)	0.009	0.12<br>(-0.96 to 1.19)	0.831	0.93<br>(-0.23 to 2.09)	0.115	-0.30<br>(-1.51 to 0.92)	0.632
Total staff in facility	0.08<br>(0.07 to 0.10)	0.000	0.07<br>(0.05 to 0.09)	0.000	0.01<br>(-0.01 to 0.03)	0.389	-0.07<br>(-0.10 to -0.05)	0.000
Observations (teams)	13,934		13,934		13,934		13,934	
R-squared	0.09		0.02		0.00		0.05	

Notes: All models show results from OLS regressions. 95% confidence intervals are shown in brackets. Observations (facilities) are clustered by census sector. The vulnerability index is standardised (i.e. coefficients show PMAQ score changes relative to a one standard deviation change in the index). The comparison group for facility type is “Health posts”.

Table A7. Association between PMAQ score and census area income for all family health teams with municipality fixed effects

	Round 1 (Nov 2011 – Mar 2013)		Round 2 (Apr 2013 – Sept 2015)		Round 3 (Oct 2015 – Dec 2019)		Difference (Round 3 – Round 1)	
	Coef (95% CI)	P value	Coef (95% CI)	P value	Coef (95% CI)	P value	Coef (95% CI)	P value
Monthly household income (in R$ 1,000)	0.25 (0.10 to 0.41)	0.001	0.35 (0.14 to 0.56)	0.001	0.26 (0.02 to 0.50)	0.034	0.01 (-0.27 to 0.29)	0.951
Proportion of census population under 5	-7.34 (-15.33 to 0.65)	0.072	4.04 (-7.10 to 15.18)	0.477	-10.88 (-23.62 to 1.87)	0.094	-3.54 (-18.20 to 11.13)	0.636
Proportion of census population over 50	-2.06 (-4.69 to 0.56)	0.123	1.94 (-1.72 to 5.60)	0.299	-3.30 (-7.48 to 0.89)	0.122	-1.24 (-6.05 to 3.58)	0.615
Facility type (health post)								
Health centre	0.36 (0.06 to 0.67)	0.021	0.45 (0.02 to 0.88)	0.038	0.36 (-0.13 to 0.86)	0.146	0.00 (-0.56 to 0.57)	0.997
Other	0.35 (-0.25 to 0.95)	0.252	0.52 (-0.32 to 1.36)	0.225	-0.38 (-1.34 to 0.58)	0.436	-0.73 (-1.83 to 0.37)	0.193
Total staff in facility	0.01 (0.00 to 0.02)	0.010	0.00 (-0.01 to 0.02)	0.666	-0.01 (-0.03 to 0.00)	0.120	-0.02 (-0.04 to -0.01)	0.006
Observations (teams)	13,934		13,934		13,934		13,934	
R-squared	0.00		0.00		0.00		0.00	
Municipalities	3,376		3,376		3,376		3,376	

Notes: All models show results from OLS regressions including municipality fixed effects. 95% confidence intervals are shown in brackets. Monthly household income is shown in terms of R$ 1,000. The reference group for facility type is “health posts”.

References

1 Buntin MB, Ayanian JZ. Social risk factors and equity in medicare payment. New England Journal of Medicine. 2017; **376**: 507–10.

2 Steinwachs DM, Stratton K, Kwan LY. Accounting for social risk factors in medicare payment. National Academies Press, 2017 DOI:10.17226/23635.
